# Management and Characterization of Abiotic Stress via PhénoField^®^, a High-Throughput Field Phenotyping Platform

**DOI:** 10.3389/fpls.2019.00904

**Published:** 2019-07-16

**Authors:** Katia Beauchêne, Fabien Leroy, Antoine Fournier, Céline Huet, Michel Bonnefoy, Josiane Lorgeou, Benoît de Solan, Benoît Piquemal, Samuel Thomas, Jean-Pierre Cohan

**Affiliations:** ^1^ARVALIS – Institut du Végétal, Ouzouer-le-Marché, France; ^2^ARVALIS – Institut du Végétal, Boigneville, France; ^3^ARVALIS – Institut du Végétal, La Chapelle-Saint-Sauveur, France

**Keywords:** field phenotyping, drought tolerance, high throughput, rainout shelters, remote sensors

## Abstract

In order to evaluate the impact of water deficit in field conditions, researchers or breeders must set up large experiment networks in very restrictive field environments. Experience shows that half of the field trials are not relevant because of climatic conditions that do not allow the stress scenario to be tested. The PhénoField^®^ platform is the first field based infrastructure in the European Union to ensure protection against rainfall for a large number of plots, coupled with the non-invasive acquisition of crops’ phenotype. In this paper, we will highlight the PhénoField^®^ production capability using data from 2017-wheat trial. The innovative approach of the PhénoField^®^ platform consists in the use of automatic irrigating rainout shelters coupled with high throughput field phenotyping to complete conventional phenotyping and micrometeorological densified measurements. Firstly, to test various abiotic stresses, automatic mobile rainout shelters allow fine management of fertilization or irrigation by driving daily the intensity and period of the application of the desired limiting factor on the evaluated crop. This management is based on micro-meteorological measurements coupled with a simulation of a carbon, water and nitrogen crop budget. Furthermore, as high-throughput plant-phenotyping under controlled conditions is well advanced, comparable evaluation in field conditions is enabled through phenotyping gantries equipped with various optical sensors. This approach, giving access to either similar or innovative variables compared manual measurements, is moreover distinguished by its capacity for dynamic analysis. Thus, the interactions between genotypes and the environment can be deciphered and better detailed since this gives access not only to the environmental data but also to plant responses to limiting hydric and nitrogen conditions. Further data analyses provide access to the curve parameters of various indicator kinetics, all the more integrative and relevant of plant behavior under stressful conditions. All these specificities of the PhénoField^®^ platform open the way to the improvement of various categories of crop models, the fine characterization of variety behavior throughout the growth cycle and the evaluation of particular sensors better suited to a specific research question.

## Introduction

The last three decades have witnessed a decline in the growth of yield trends (AGRESTE, 2018) which has been attributed to climate change rather than to breeding or agronomical causes (Brisson et al., 2010). Climate changes are in general unfavorable to cereal yields in temperate climates because of higher heat stress during grain filling and longer drought during stem elongation. Regional model simulations for climate change in mid-Europe agree on an occurrence of higher variability in rainfall in the coming decades, with an increased risk of water shortage during summers (Jacob et al., 2013). At the same time, water resources available for agricultural irrigation will be reduced or maintained in the best scenarios (Barros et al., 2014). Moreover, the crucial role of nitrogen on production and quality of the harvested organs (Jensen et al., 2011) coupled with the potential impact of nitrogen losses on the environment (Galloway et al., 2003; Sutton et al., 2011) lead to an increasing concern about the improvement of the nitrogen use efficiency of the agricultural systems. This perspective enforces the need to design strategies and tools that combine novel crop genotypes and adapted crop management techniques to assist agriculture in facing major challenges, such as increasing rainfall variability and the reduced availability of fertilizers (Wreford et al., 2010).

While genomic capacity encountered a breakthrough in 2010, phenotyping capacity has become the major limitation in breeding programs aimed at building genotypes that maintain or increase crop performance under climate changes and reduced inputs (Furbank and Tester, 2011). In field conditions, conventional phenotyping represents high investment, it is laborious, mainly destructive, and could weaken significance or precision of results from consolidated large experimental reliant networks. Numerous measurements on a broad genetic diversity panel are now perceived as key levers of genetic advances and lessen the potential added value of modern techniques such as marker-assisted selection, or genomic selection (Araus et al., 2018). To address this issue, significant efforts have been made to encourage the capacities of multilevel phenotyping in worldwide initiatives and dynamics, creating networks and communities. The acceleration of instrumentation (Reynolds et al., 2018; Roitsch et al., 2019) and sampling capacities (Pieruschka and Schurr, 2019) has opened the way for further investigation in epigenetic mechanisms and plant physiology with the possibility of building advanced digital models of plant physiology which underpin research and decision support services (Jiang et al., 2018; Tardieu et al., 2018; van Eeuwijk et al., 2018).

The French Plant Phenotyping Network^1^, PHENOME-EMPHASIS/France, funded by the French National Research Agency (ANR), and lead by the National Institute of Agricultural Research (INRA), provides French researchers with up-to-date, high throughput infrastructures and methods allowing the characterization of different species under scenarios associated with climate change. The project aims (i) to build and upgrade highly instrumented platforms in nine French sites able as a whole to grow the most common crop species under a large range of environmental conditions, (ii) to develop new sensing technologies, associated with advanced data processing and management, (iii) to disseminate the newly developed techniques and methods within the French phenotyping community (breeders, technical institutes, and public research groups) and (iv) to enhance the emergence of French SMEs involved in developing phenotyping methods.

The PhénoField^®^ platform is management by the applied research institute ARVALIS and is part of the PHENOME-EMPHASIS/France project. It is an original field phenotyping platform enabling the design of a large range of drought and nutrient availability scenarios and the fine characterization of crop functioning as a response to these abiotic stresses. This accurate monitoring of both growing conditions and crop growth in the field is a key to improving the analysis of genetics × environment interactions and to identifying genotypic markers associated with favorable crop behavior. To this end, the PhénoField^®^ platform manages a moving rainout shelter and irrigation systems that allow the application of different field drought conditions (since 2015), all the while coupled with environmental sensors to control drought stress environments. PhénoField^®^ uses high-throughput phenotyping technologies set (validated and innovative sensors) on an automated gantry (since 2017), allowing frequent and non-invasive high-resolution measurements of the canopy. Its location at Ouzouer-le-Marché/Beauce la Romaine (41), central France, makes PhénoField^®^ representative of irrigated crop farms of the Beauce area with the capability of studying large genotype panels of various species (bread wheat, durum wheat, corn, etc.).

2017 was the 1st year offering advanced capability on PhénoField^®^. During this crop season, PhénoField^®^ carried out a bread wheat field trial in the framework of the BREEDWHEAT project^2^, the purposes of which are to strengthen the competitiveness of the French wheat breeding sector and address the societal demand for sustainability, quality, and safety in agricultural production. The BREEDWHEAT project aims to develop new breeding methodologies and use unexploited genetic resources to identify and combine alleles of interest into new ecological friendly varieties adapted to climate changes, including the enhanced adaptation to increasing biotic and abiotic stresses. The trial hosted in 2017 in the PhénoField^®^ platform as a node of a trial network, focused on water and nitrogen stresses and their interaction with genotypes. Indeed, one of the key drivers of yield gap mitigation and reduction is reducing the reliance on nitrogen fertilizers (Hawkesford, 2014) or a better Nitrogen Use Efficiency (NUE) and Water Use Efficiency (WUE) through improved understanding of respective and crossed mechanisms driving these parameters in plain field conditions (Fischer and Connor, 2018). It evaluates the functioning of 22 bread wheat varieties, representative of the last three decades of genetic innovation, under nitrogen and water deficit. Phenotyping data were acquired during the growing season using conventional and innovative techniques.

This paper presents the PhénoField^®^ phenotyping platform. We first evaluate its capacity to control crop-growing conditions and potential biases due to the presence of mobile shelters. Related to this, a set of tools and procedures have been assessed to finely monitor and record weather data and soil water status; then, the high throughput phenotyping system is described. It includes automated sensing tools and the related data processing methods.

As an example here, results obtained during the 2017 BREEDWHEAT experiment have been analyzed to answer two questions: Is the platform able to generate the desired abiotic-stress scenarios? How is the phenotyping system able to reveal differentiated bread wheat behaviors amongst water deficit conditions, nitrogen deficit conditions or a studied genotypic panel?

## Materials and Methods Developed on the PhénoField^®^ Platform

**FIGURE 1:**
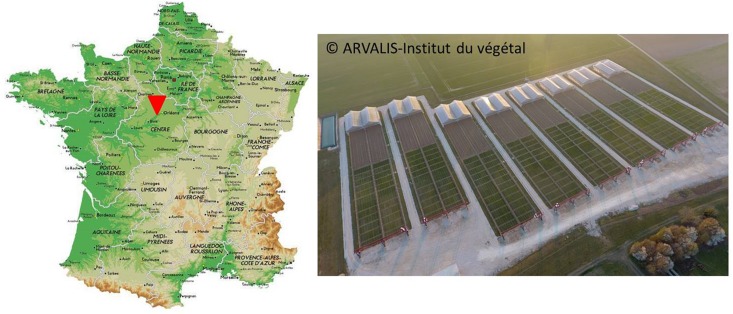
Location of PhénoField^®^ platform in France, near Orléans, and aerial view of the 8 rainout shelters and gantries around wheat field trial (*GPS coordinates: 47°53*′*01.6*′′*N 1°31*′*16.4*′′*E*).

The PhénoField^®^ platform is located at Ouzouer-le-Marché/Beauce la Romaine (41) in Beauce region, one of France’s most productive agricultural areas hosting a wide variety of cultivated crops (Figure 1). It was implemented in 2013 on 7.5 hectares of farmland and is equipped with 8 moving rainout shelters, environmental sensors and high throughput phenotyping facilities. A web-based user interface, named PhenX (Piquemal, 2017), allows data visualization and downloading.

### Managed Environment

#### Mobile Rainout Shelters

**FIGURE 2:**
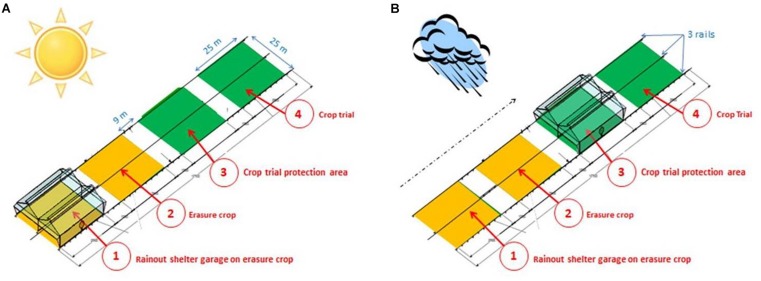
Four crop growth areas to ensure a correct crop rotation every year (switch between green area and yellow area) and to avoid the effects of drop shadows on the crop trial (at least 35 m). **(A)** No rain position; **(B)** position during a raining event.

Each rainout shelter covers 655 m^2^ (about 25 m × 25 m), and is equipped with an automaton controlling its movement. The central controller is linked to a rain contact sensor and sonic anemometer to, respectively, trigger the movement of the rainout shelters and secure the infrastructure (in case of strong wind). Each of the 8 rainout shelters is seated on three 150 m-long rails in order to move them from a garage position (when it does not rain Figure 2A) to a rain controlled position (when raining Figure 2B). The rainout shelters are arranged along 4 cropping areas to ensure adequate crop rotation every year with, generally, 2 cropping areas with the trial crop (Nos. 3 and 4, Figure 2) and the 2 others with an “erasure crop” (Nos. 1 and 2, Figure 2) including one area for park position. This experimental design allows firstly, the rainout shelter to be stationed at 43 m further than the trial area, thus avoiding the drop shadow during non-rainy periods and secondly, the rain- controlled position to operate in the same area every 4 years. To maintain this efficient crop rotation, the erasure crop must be chosen according to the species studied. Field trial species on PhénoField^®^ are therefore discussed nearly 18 months before trial implementation.

The 8 rainout shelters protect 384 m × 6 m field trial microplots (1 m × 6 m; Figure 1-rigth part). Each shelter, formed by 2 spans, manages 48 microplots with experimental modalities applied per group of 24 microplots at span scale. For crop management, PhénoField^®^ is equipped with a special spray boom to treat one span from the central aisle between rainout shelters and so avoid affecting soil compaction with machinery under rainout shelters. Thus, nitrogen or crop protection cross-factors are set up at span scale.

The eight rainout shelters are equipped with their own individual irrigation networks, allowing precise management of the water supply in protected plots. Two booms per rainout shelter allow up to 16 different irrigation modalities. Hence PhénoField^®^ can perform between 1 controlled irrigation modality on 384 microplots to up to 16 modalities of 24 microplots. Using the rainfall area (when rainout shelter parking is on area no. 1 or no. 4), PhénoField^®^ allows a 768-microplot field trial. Roof gutters collect the rain water used for irrigation. This whole infrastructure allows crops to be subjected to a pre-determined duration of water stress at any desired period of their cycle.

#### Soil Characterization

The management of crop water stress implies precise soil characterization established with measurements of soil resistivity and water holding capacity (WHC) on the entire PhénoField^®^ platform.

The electrical resistivity of the soil is a physical quantity related to the soil’s intrinsic characteristics (clay content, texture, water content, depth, etc.), with the higher values representing soil resistance to current flow. This magnitude of soil resistivity is measurable at high resolution and allows, for some types of soil, to extrapolate geographically located measures of water holding capacity (Michot et al., 2003). Soil resistivity measurements were performed by using the Automatic Resistivity Profiling (ARP) method which uses a multi-electrode system towed by a quad bike for rapid data acquisition (Dabas, 2008). One pair of electrodes was used for electric current injection and 3 pairs were employed for measurements in order to investigate the soil resistivity between 0–50, 0–100, and 0–200 cm. ARP measurements were performed in September 2011 over the entire farmland to acquire soil-resistivity data used to get a global view of the agricultural plot and optimize rainout shelter locations on the farmland.

The soil observed on the agricultural field is Beauce clay loam with a loamy clay texture on calcareous Beauce rock. Samples have been taken to determine the WHC/cm of the different types of soil layers based on granulometric analyses. Spatialization of soil layer thicknesses was performed at the beginning of platform construction by using the 1,100 pits opened for pouring concrete pads, from 0 to 1.5 m for each pit. In more detail, we measured four kinds of soil thicknesses: LA which corresponds to plowed horizon, S which is cambic horizon, C1 and C2 which correspond to calcaric material (C1 is cryoturbed limestone and C2 is sandy calcaric material). Based on these data combined with pF data for each horizons characterized, soil mapping was generated with krigeage models providing soil layer thicknesses but also WHC estimation at every point of the platform. As microplots are georeferenced, an estimation of the WHC was performed for each of them by computing the mean of the WHC points contained in the corresponding area. Data management was operated by PostgreSQL software, a relational database management system extended with PostGIS software to add support for geographic objects.

#### PhénoField^®^ Rainout Shelter Efficiency Characterization

Evaluation of the shelters’ capacity to efficiently intercept rainfall was evaluated in 2017 by measuring precipitation along transects of crops protected by the shelters. Seven pluviometers were installed at equivalent distances (6 m) with five installed under the area protected by rainout shelters and two others installed on each side of this protected area. Pluviometers were positioned between each microplot-line.

Possible side effects on photosynthetically active radiation (PAR) and temperature were also assessed in 2017 by using, respectively, two quantum sensors (SKP215, Campbell Scientific) and two thermocouples (T109, Campbell Scientific). One of each sensor was set up in the center of the area protected from rain and the other one outside the protected area. Comparisons of air temperature and PAR inside and outside the shelter-protected area were performed by measuring the cumulative PAR and degree-day over the period of crop protection. The cumulative daily light was calculated as the sum of PAR received each day by the crop (in μmol.m^–2^) and the cumulative degree-day as the mean of maximum and minimum daily temperatures added up over the day with the 0 value corresponding to the 1st day of rain interception.

### Stress Control

#### Environmental Monitoring

The meteorological conditions on the PhénoField^®^ platform are monitored by a weather station measuring air temperature, atmospheric pressure, diffuse radiation, relative humidity, wind speed and direction in 15-min steps. The soil humidity and soil water tension at 30, 60, and 90 cm deep are recorded in control plots under each rainout shelter.

Irrigation management was realized using Irrinov^®^ method (Bouthier et al., 2003). Irrinov^®^ is an online free irrigation tool based on tensiometer measurements^3^. It gives tension thresholds above which farmers have to irrigate for Watermark^®^ tensiometers at 30 and 60 cm deep in a given situation (3 tensiometers are placed at each depth). This method has been developed by Arvalis and its partners for different regions in France. Thresholds depend on four main variables: climatic demand, soil type, crop and period between two irrigations (parameter which depends on farmer irrigation equipment). It is made for farmers to manage irrigation in its tactic phase. Thresholds were determined with field trials in different French regions. For PhénoField^®^, we chose Irrinov^®^ method adapted to French region Centre, for a deep soil and for wheat. In these conditions, 30 cm-tensiometer threshold is 100 cbar and 60 cm threshold is 80 cbar before last leaf spread growth stage (Z39) and 100 cbar after. To manage well-watered irrigation during the BREEDWHEAT trial, we decreased thresholds to bring water in the field to 80 cbar also after Z39 to be sure plants do not suffer from water stress in well-watered conditions (WW). This method allowed us to consider that under 120 cbar threshold at 60 cm wheat is not suffering from water stress. Unfortunately, this kind of probe cannot record signal higher than 200 cbar and they don’t measure soil humidity directly.

It was therefore necessary to have other kind of sensors to measure soil humidity. To do that, soil humidity was measured with Time Domain Reflectometry probes (TDR-TRIME-PICO 64) installed at 30, 60, and 90 cm deep in control plots under each shelter. This type of probe has the advantage of being buried for 5–10 years without being moved. To position them deep in the soil it was necessary to make small trenches and TDR probes are known to be very sensitive to their immediate environment (air, ground contact with the pins, pebbles, etc.) so they must have been calibrated with a series of five gravimetric measurements performed every 2 months. Gravimetric water content was determined by measuring the weight of freshly collected soil (near each probe) and a soil sample oven-dried at 110°C over 48 h (see Supplementary Figure S1).

In addition to measurements by the probes on control plots, soil nitrogen content was measured following a colorimetric method using a KCL extraction on samples taken before sowing, at the end of winter and after harvesting.

#### CHN: A Model to Quantify Abiotic Limiting Factors

Agro-meteorological conditions are incorporated into a dynamic crop model (called ‘CHN’) used to estimate crop growth, manage crop practices and evaluate crop responses to water and nitrogen shortage (Soenen et al., 2016). This model calculates the daily flow of carbon (C), water (H), and nitrogen (N) between the soil, atmosphere and plant compartments at a daily time stage during a cropping season (see Supplementary Figure S2). Soil and Atmosphere compartments are connected to databases using, respectively, different soil characteristics in France and daily weather data (over 250 sites with 25 years data; Soenen et al., 2016). The plant compartment is based on the Monteith approach (Monteith, 1994): leaf growth is modeled and intercepts radiation that is converted into biomass. The Green Plant Area Index (GPAI), transpiration and biomass are affected by water and nitrogen deficiency, according to functions of stress response developed by Sinclair (Sinclair, 1986). These functions provide a stress factor between 1 (minimum stress) and 0 (maximum stress), that is used to slow down the potential growth and transpiration. Links between soil–plant–atmosphere compartments are the background of a model of the water and nitrogen balance. Coupled with frequentist weather forecasts, CHN outputs are complementary to probe measurements for water and nitrogen input management to follow-up the situation of each shelter line each day.

#### Trial From the BREEDWHEAT Project

In 2016–2017, a bread winter wheat field trial was conducted for the BREEDWHEAT project. It aimed to evaluate 22 varieties, mutual to other field experiments and known for their diversity of responses to different stresses, especially differing in behavior to nitrogen- and water-stressed conditions. Using six of the eight rainout shelters from PhénoField^®^, it was implemented with a double split-plot design in order to group water management treatments under rainout shelters and two nitrogen fertilization levels per rainout shelter (one per span) (see Supplementary Table S1).

The two water management treatments consisted of:

1. Well Water conditions (called “WW”) without rain interception and good irrigation practices (following the IRRINOV^®^ method and CHN model).
2. Water Deficient conditions (called “WD”) with the interception of rainfall in the period between the first node and grain filling growth stages (from 22nd February to the 25th June) and irrigation occurring only to allow nitrogen uptake from fertilizer.

Each water management treatments was applied to 3 shelters and separated per span so as to have two nitrogen levels:

1. With optimum nitrogen supply (receiving a total 132 kg N.ha^–1^, called “N+”).
2. Without N supply (called ‘N0’).

The 22 bread winter wheat varieties were randomized under each span to evaluate their agronomic performances under these 4 modalities with 3 biological replicates (22 varieties × N+/N0 × WW/WD × 3 replicates). One control variety, APACHE, was triplicated in order (i) to perform destructive measurements, (ii) to grow above the soil tensiometers and TDR probes and (iii) to measure non-destructive variables and yield components.

The sowing was performed on 2016 October 20th and the harvest occurred on 2017 July 11th for the WD and 2017 July 18th for the WW due to differences in maturity stages. Good agricultural practices in plant protection were performed to avoid weeds, pest and disease effects on the trial. Agronomic traits were measured on each microplot:

1. Phenology: sowing date, emergence date, heading date, flowering date, harvest date.
2. Yield components: plant density (plants.m^–2^), spike density (spikes.m^–2^), dry matter grain yield (GY, t.ha^–1^), thousand Kernel Weight (TKW, g), grain protein content (P, %). Nitrogen Grain Quantity (Nabs, Kg.ha^–1^) was calculated using GY^*^P/5.7.

On check plot, above ground biomass and nitrogen content (based on the Dumas combustion method) were measured at flowering stage and also at maturity stage, distinguishing straw and grain to measure harvest index and nitrogen harvest index.

Statistical analyses were conducted using R studio software version 3.4.4 (R Core Team, 2017). The effects of water and nitrogen stresses and variety on agronomic variables were assessed with variance analysis with these three factors and their interactions.

### High Throughput Phenotyping Data

#### Phenotyping Gantry and Sensor Bay

A set of eight fully automated phenotyping gantries were installed over the moving rainout shelter rails in order to acquire frequent crop canopy measurements via remote sensors, thus ensuring non-invasive measurements and the collection of a large amount of phenotyping data. Each 25 m wide gantry is able to lift a payload at a 6 m height, allowing data acquisition on any type of crop, even tall maize cultivars. These data are obtained with the sensors installed on a high throughput phenotyping bay, mounted on the gantries during experimental campaigns. It allows smoothed screening from 0.1 to 3 m.s^–1^ and centimetric controlled repositioning of canopy sizing from 0 to 3 m. Each sensor head can carry a set of sensors with no limit of power consumption and up to 150 kg. New sensor installation is possible thanks to its payload capacity and its agile interfacing. An open robotic operating system (ROS; Quigley et al., 2009) was used to allow interfacing of several sensors and the management of spatial and temporal sampling on each microplot.

**FIGURE 3:**
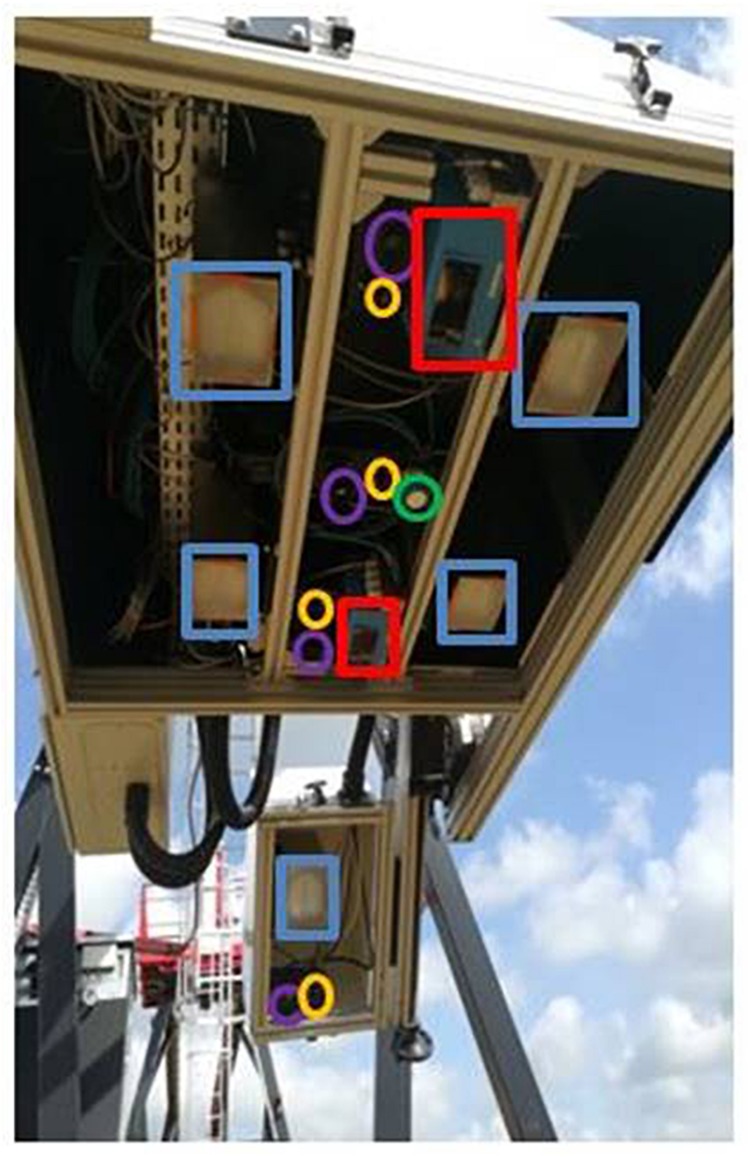
Bay carrying the sensors with 2 angles of view. The shapes show the 2 LIDARs (in red), the 4 cameras (in purple), the 4 spectroradiometers (in yellow), the flashes (in blue) and the telemeters (in green).

Two identical phenotyping bays are currently used on the 8 gantries to carry several types of optical sensors. The position of the sensors was optimized in order to spatially sample the area of interest and allow intra-plot borders removal. Each sensor bay had 2 measuring viewpoints: an optical head at the vertical of the vegetation (nadir) and an angular view positioned at 45°. The two sensor bays also included 4 xenon flashes to allow active measurement and standardization of daily radiation acquisition. Flashes are distributed on the vertical and inclined bays to ensure good illumination homogeneity over the camera and spectrometric field of view. An ultrasonic actuator coupled to the robotized gantries was used to estimate the height of the crop canopy and automatically set up distance to target in a closed loop control. The available sensors were (Figure 3):

1. RGB industrial cameras (VLG40c, Baumer, Ger; 2044 pixels ^*^ 2044 pixels for 28° optical aperture) to ensure the measurement of the fraction cover, green fraction, green plant area index and average leaf index. The resulting fields of view in the object plane measure 60 cm^*^60 cm corresponding to a resolution of 0.29 mm per pixel at a 1.5 m distance. The typical configuration for wheat is a set of 3 RGB cameras (two cameras viewing at 0° from vertical and one at 45°).
2. A VIS-NIR spectroradiometer (MMS1, ZEISS, and Ger) with a measurement range of 380–1,100 nm covered by 256 pixels feed by a large core optical fiber of numerical aperture 0.2. The resulting full field of view at a sensing distance of 1.50 m is 60 cm. It allows the quantification of the light reflected by the crop canopy and the biochemical composition of plants via vegetation indices traits. The typical configuration for wheat is 3 spectroradiometers (two sensors viewing at 0° and one at 45°).

LiDARS (LMS 400-1000, Sick, and Ger.) scan at 650 nm with detection ranging from 70 to 300 cm. This sensor allows the characterization of the 3D structure of the canopy and the estimation of plants height. The acquisition is continuous for a given microplot with a scanning frequency of 290 Hz and an angular step of 0.2°. The resulting transversal and longitudinal resolutions are, respectively, of 5 mm and 1 mm for a scanning speed of 0.3 m.s^–1^ at a sensing distance of 1.5 m. The typical configuration for wheat is 2 LiDARS (both viewing at 0°).

#### Acquisition, Data Calibration

The level and stability of the sensing chain including illumination, geometric configuration, light transmission and sensor response functions were set up to optimize signal to noise ratio of low level data and were documented. Every day of acquisition, controls were performed systematically against a secondary calibration surface and tracked by the National Institute of Standards and Technology (NIST) through Spectralon^®^ (Labsphere, NH, United States) in accordance with good practice for uncertainty management (GUM). This data were used to correct the white balance of RGB cameras and to calculate physical units of reflectance. Acquisition was optimized to maximize the sampling within the microplot and to allow full acquisition of the platform in 1 day with a two-sensors bay. During the 2017 campaign an operation speed of 0.3 m.s^–1^ was chosen allowing three acquisitions of each RGB image and of VIS-NIR reflectance measurements. LiDAR acquisition was carried out all over the plot area.

#### Processing and Interpretation

For RGB cameras, a white balance process was first applied to adjust intensities of the red, green, and blue channels at a same intensity on a reference gray panel. This standardization was important for a robust color based image analysis.

The first use a RGB images was the calculation of the green cover fractions (GCF) at 0° and 45°. A support vector machine algorithm trained on a reference dataset, was used to classify for each image the green and non-green pixels and determine the percentage of green elements for a given viewing angle (Dutartre et al., 2015).

The green cover fractions at 0° and 45° were used to estimate the Green Area Index (GAI) and Average Leaf Angle (ALA). Both variables were estimated by inverting a simple Poisson model using the measured gap fractions Po, calculated as (1-GCF). The model used to relate Po to GAI is:

$$Po\left(\theta p\right)=e^{-\frac{G\left(\theta p,\theta l\right)}{\cos\left(\theta p\right)}.GAI}$$

Where θp is the viewing angle, θl is the mean leaf angle and *G*(θp,θl) is the function that expresses the projected area of the leaves for a particular configuration. We assumed that the G function follows an ellipsoidal leaf angle distribution (Campbell, 1986, 1990). The model was inverted using a look up table minimization procedure (Weiss et al., 2000, 2004) to retrieve the more likely combination of GAI and ALA.

For spectroradiometers, a calibration measurement on a spectrally characterized reference surface was done before each data acquisition session. The reflectance at the crop level was then obtained by dividing the canopy reflectance by the calibration measurement. At the plot level, the averages of the normalized reflectances were computed.spectroradiometer Satisfactory signal to noise ratio (giving a threshold of 20) ranges from 450 to 820 nm. Then physically expressed reflectance was sampled by Gaussian filters corresponding to bands needed for calculation of the vegetation indexes. Three vegetation indexes from remote sensing literature were selected for their asserted link with different phenological aspects of the aerial part of monitored crop. The vegetation indices calculations were made with a 3 nm Full Width Half Maximum (FWHM) for all bands. The Normalized Difference Vegetation Index (NDVI; initially proposed by Rouse et al., 1974) is a basic and robust indicator of the amount of vegetation in the field. It correlates firstly with the cover fraction in the direction of sight and secondly with GAI.

NDVI=R800-R670R800+R 760

The Meris Terrestrial Chlorophyll Index (MTCI) was initially proposed by Dash and Curran (2004) in order to extend the accuracy of red-edge position estimation on crops with higher chlorophyll content. Initially designed to exploit Medium Resolution Imaging Spectrometer built into the platform, it showed a better capacity than other red-edge based indexes to estimate chlorophyll content for higher LAI values when the canopy is closed.

$$MTCI=\frac{R754-R709}{R709-R681}$$

The Modified Chlorophyll Absorption Ratio Index (MCARI2) proposed by Haboudane et al. (2004) is a non-dimensional empirical index targeting green LAI of crop canopies for precision agriculture purposes. It is tailored by modeling in order to minimize the effect of leaf chlorophyll content on the prediction of green LAI. By construction, it is a non-normalized index and its value is sensitive to reflectance spectra intensity contrary to previous indexes.

$$MCARI2=1.5\times \frac{2.5\times \left(R800-R670\right)-1.3\times \left(R800-R550\right)}{\sqrt{{\left(2\times R800+1\right)}^{2}-\left(6\times R800-5\times \sqrt{R670}\right)-0.5}}$$

Plant height (cm) was estimated from the analysis of the 3D point cloud generated from the combination of the LiDAR scans of height (z) and the (x,y) positioning of the sensor, recorded by the gantry’s encoders. The plot mean height was calculated using the algorithm developed by Madec et al. (2017). It first consisted in clustering the point cloud to separate the ground from the vegetation. The maximum peak in the z-distribution of the non-vegetation points was assigned as the ground level. This distance was subtracted from the 3D point cloud resulting into a distribution of the height values. The height of the canopy was then defined as the height value corresponding to 99.5% of the cumulated height distribution of the vegetation points.

After statistical and physical validation against expected intermediate values and validation of biophysical values in order to control non-divergence in case of inversion techniques, data were uploaded and shared through a dedicated database named PhenX (Piquemal, 2017) allowing statistical analyses following good practices in plant experiments. The temporal evolution of each estimator could then be analyzed by fitted parametric models. Deduced parameters and the integration of deduced models over specific phenological periods could then produce meaningful indicators of ideotype-variability (as the area under the curve).

## Results: Study in 2017

### The PhénoField^®^ Platform Characterization and Environmental Monitoring

**FIGURE 4:**
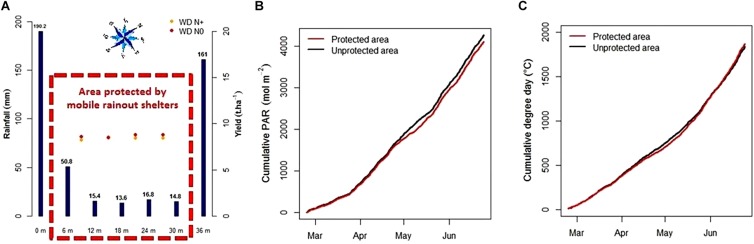
Precipitation levels (mm, blue bars) and yield mean (t/ha, red dots for N+ environment and yellow dots for N0) of the 4 plot-lines using their location from the beginning of the protected area (m, red dotted lines) in water-deficit condition **(A)**, cumulative daily PAR **(B)**, and cumulative degree days **(C)** during the crop protection from rain (from 02/23/2017 to 06/25/2017).

The use of the mobile rainout shelters from 23rd February to 26th June 2017 reduced detected precipitations under the protected area for the WD environment. During these 4 months, the sum of precipitations collected by the 2 pluviometers outside the protected area reached 161–190 mm (Figure 4A) and was around 15 mm under it, except for the first pluviometer under the protected area in a south west position that received 50 mm (Figure 4A). This surplus of precipitation in this position was probably due to precipitations brought by the prevailing southwesterly winds but did not significantly affect the yield of the first plot under WD condition (red and yellow point, Figure 4A).

During the period of use of the mobile shelters, PAR and air temperature were also affected with a 49% linear decrease in the PAR and a 0.85°C global increase in the air temperature (Supplementary Figure S3). These effects only occurred during protection with the rainout shelters which represented less than 8% of the 4 months of rain interception including half of this time at night. As a result, accumulation of the daily PAR was reduced by 3.5% and, by contrast, it led to a 1.8% increase in the cumulative degree days (Figures 4B,C).

### Stress Management and Indicators

**FIGURE 5:**
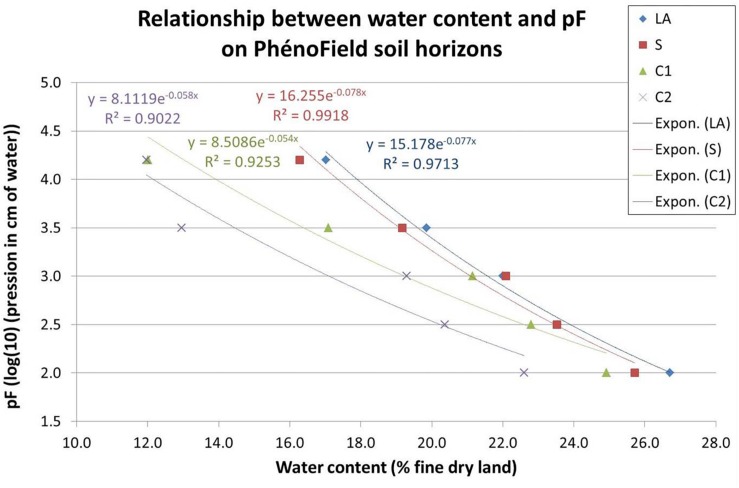
Relationship between water content and pF on PhénoField soil horizons; LA: plowed horizon, S: cambic horizon, C1 et C2: calcaric material (C1: cryoturbed limestone; C2: sandy calcaric material).

For each soil layers, LA: plowed horizon, S: cambic horizon, C1 and C2: calcaric material (C1: cryoturbed limestone C2: sandy calcaric material), Figure 5 shows pF curve results. PhénoField^®^ soil water content could vary between 12 and 27%.

**FIGURE 6:**
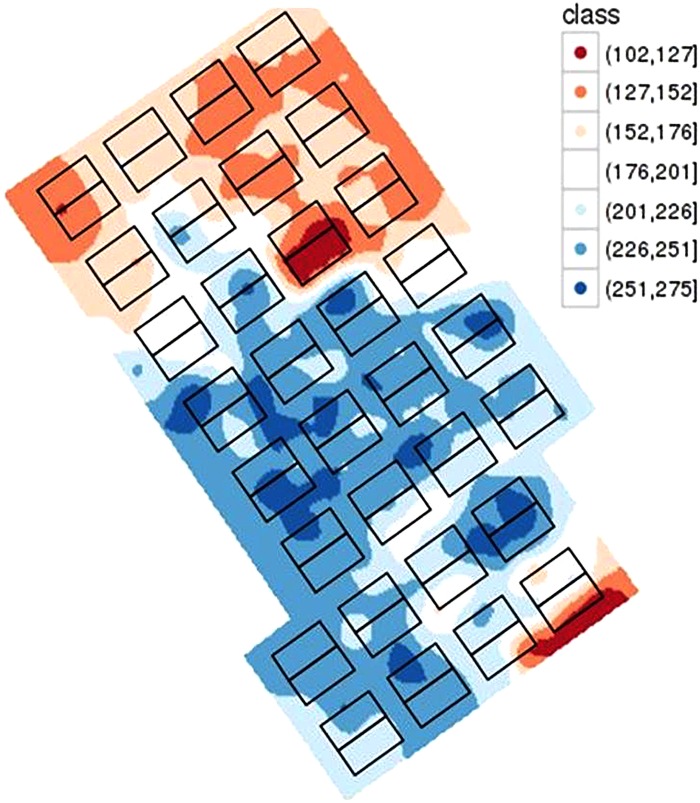
Characterization of the soil water holding capacity (mm) on the PhénoField^®^ platform with rectangles representing a span in each of the eight shelters and for the four positions.

**FIGURE 7:**
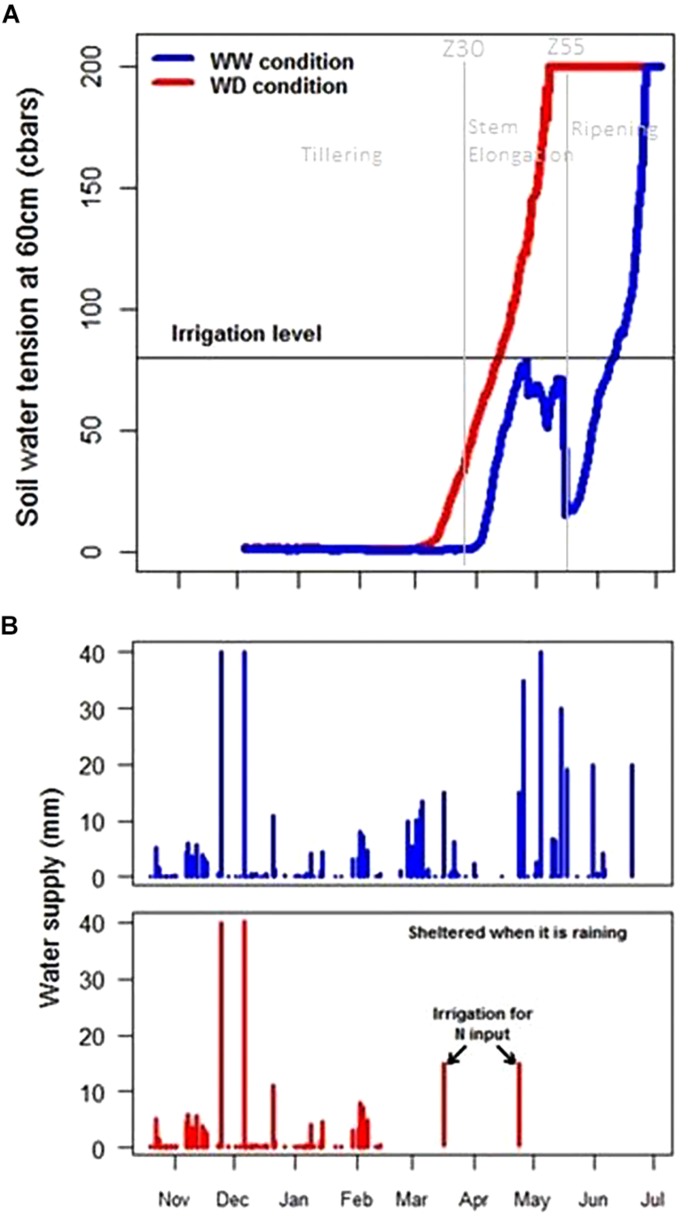
Soil water tension at 60 cm **(A)** and water supply **(B)** in well-watered (blue) and water-deficient conditions (red) during crop growth.

Mapping the soil characteristics revealed an important variability of the WHC over the site with a WHC varying from 102 to 275 mm (Figure 6). On the crop trial protection area, during 2017, WHC varied from 133 to 263 mm. Application of WD conditions on such soil variability was monitored by tensiometers. Before wheat trial protection by mobile rainout shelters on 23rd February, the WHC was filled and thus contained a mean water quantity of 184 mm. Rain interception led to a rapid increase of the soil water tension at 60 cm in WD conditions at the end of March (Figure 7A, red curve), 3 weeks earlier than in WW conditions. The threshold of 80 cbars to trigger irrigation and avoid water stress was reached several times and induced irrigation in the WW conditions and hence led to a rapid decrease in the soil water tension (Figure 7A). The threshold of 120 cbars which expresses no water stress for wheat in PhénoField^®^ conditions, was reached at the end of April (between 04/25/2017 and 05/05/2017) for the WD conditions (Figure 7A).

The linear relationship between the gravimetric measurement and TDR values allowed good calibration of the TDR probes (Supplementary Figure S1). This calibration performed in, 2017, needs to be confirmed through other soil samples in the coming years. As such, these fixed probes will estimate control-plot plants’ water consumption (mm/day).

A summary of rainfall, irrigation and nitrogen fertilization per month is reported (Supplementary Table S1). Two irrigations were performed on WD during March and April to increase nitrogen uptake just after the fertilizer application. These irrigations represent a small quantity of water (15 mm) and have no impact on soil tension at 60 cm depth as shown in Figure 7A. In total, according to weather station and irrigation data, the wheat received 478 mm of water input in WW conditions and only 211 mm in WD conditions (Figure 7B).

**FIGURE 8:**
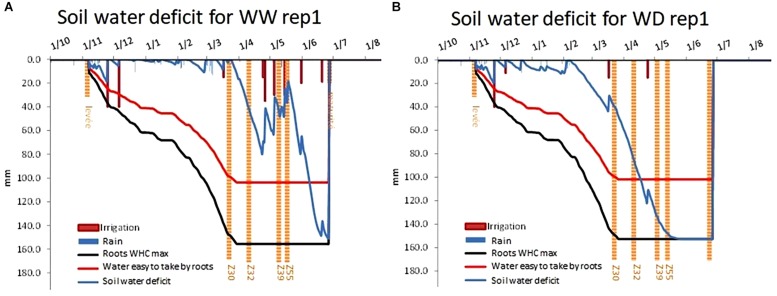
Soil water deficit level for well-watered conditions **(A)**, for WD water-deficit conditions **(B)** using CHN Model outputs.

Combining the soil mapping and the weather measurements, CHN model helped us to monitor daily soil water deficit under each rainout shelter (Figure 8). Considering repetition one of BREEDWHEAT trial which was located under shelters number 1 and 2 as an example, CHN model simulated water available to plant roots during plant cycle. Here we can see that at this place on PhénoField^®^ platform, WHC was about 155 mm. These results are consistent with probe measures as described above.

**FIGURE 9:**
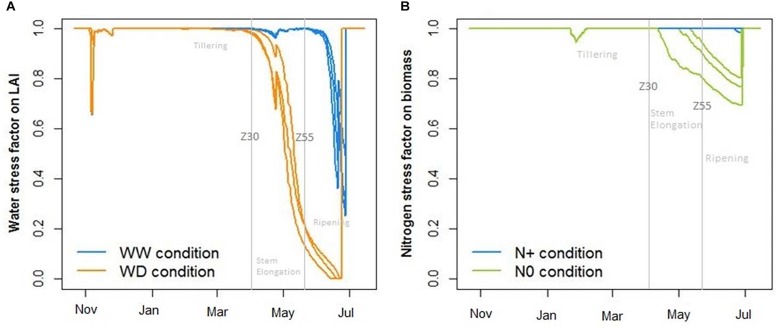
Impact of water **(A)** and nitrogen **(B)** deficiency on, respectively, leaf area index (LAI) and total biomass where WW, well-watered conditions; WD, water-deficient conditions; N+, optimum nitrogen supply; and N0, without nitrogen supply. Stress indicators calculated with the crop model CHN.

In another way, the CHN model allowed us to calculate abiotic stress factors induced on crops per replicate (Figure 9). The water stress factor stimulated by CHN showed an important effect of water deficiency on the LAI, which began at the end of April with slight heterogeneity between replicates (Figure 9A). With regard to the nitrogen stress factor, it affected the wheat biomass with greater heterogeneity between replicates (Figure 9B).

### Stress Impact

**FIGURE 10:**
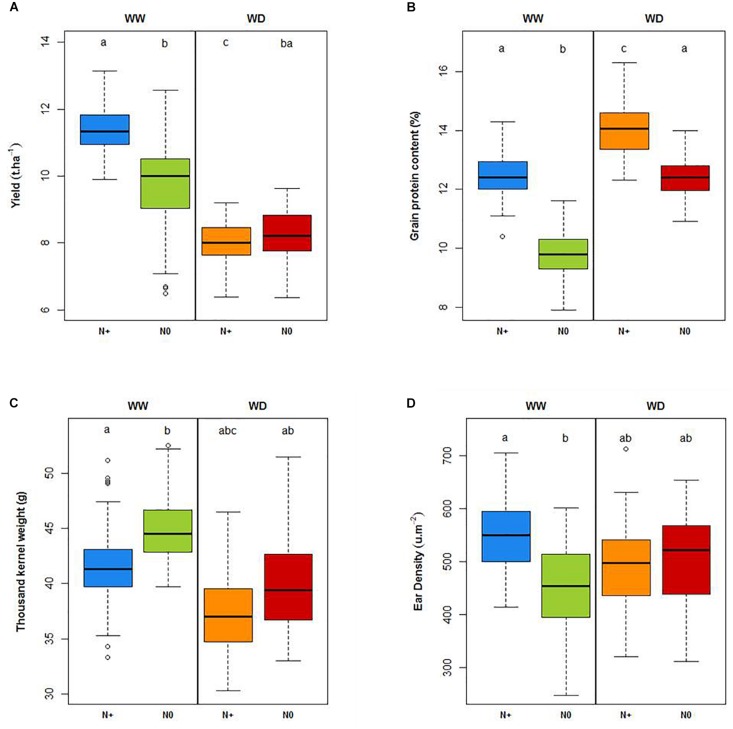
Yield (t.ha^–1^; **A**), grain protein content (%; **B**), thousand kernel weight (g, **C**) and ear density (μ.m^–2^; **D**) under well-watered (WW) and water deficient (WD) conditions with and without nitrogen (N+, N0). Letters represent significant differences (Tukey *post hoc* test, *p* < 0.05).

With regard to variety behavior, both water and nitrogen deficiency significantly reduced the yield (Figure 10A). The WHC was filled at the end of February. Up until the end of March (Z30 stage), this 184 mm of water allowed crop growing in optimal conditions without watering. Tilling was carried out in good conditions with good root development. The lack of 267 mm of water reduced the yield from 11.4 t.ha^–1^ in WW N+ conditions to 8 t.ha^–1^ in WD N+ condition. This water deficit was rather strong and resulted in a 30% decrease in yield. Along the same lines, yield from WW N+ conditions decreased to 9.7 t.ha^–1^ in WW N0 conditions (15% decrease in yield). The interaction of both stresses (WD N0) led to a yield of 8.3 t.ha^–1^, which is not significantly different from the WD N+ conditions. During this experiment, yield potential under water deficiency was reduced by 30%; consequently, even if we did not bring nitrogen input on the WW N0 condition, soil nitrogen amounts supplied by the soil were sufficient to maintain an equivalent yield between WD N + and WD N0. Yield reduction between WW N+ and WW N0 is linked to a significant ear density reduction due to nitrogen stress (Figure 10D) with partial compensation linked to an increase in thousand kernel weight (Figure 10C). A yield decreasing due to water deficit with no change in supplied nitrogen logically caused an increase of the protein concentration in the grain (Figure 10B). Significant differences of agronomical traits under water and nitrogen deficiency are also shown in Supplementary Table S2 (yield, grains protein content, thousand kernel weight Plants density, Grains.m^–2^ calculated with three-factor ANOVAs).

**FIGURE 11:**
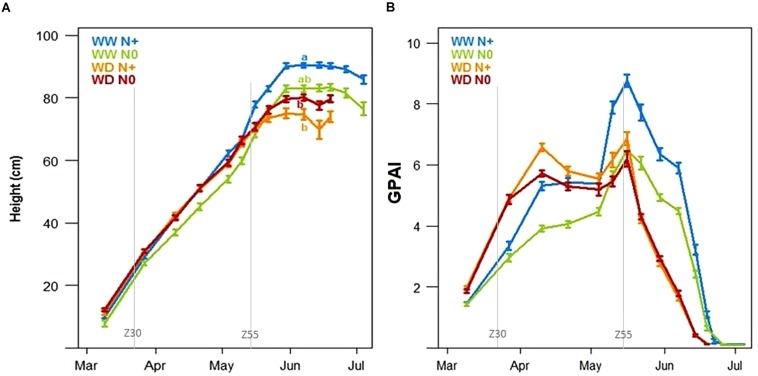
Mean of wheat height (cm) **(A)** and green plant area index **(B)** during the growing season under well-watered conditions with and without nitrogen input (WW N+ and WW N0) and water deficient conditions with and without nitrogen input (WD N+ and WD N0, *N* = 44, ±SE). Letters represent significant height differences on 30th May (Tukey *post hoc* test, *p* < 0.05).

Looking at plant height based on LIDAR data, both water and nitrogen stresses significantly reduced the wheat height at the beginning of the grain-filling period (Figure 11A). In this trial, water deficiency, during the stem elongation stage (between Zadok 30 and Zadok 55), reduced maximum plant height by 15 cm (WW N+ compared to WD N+ conditions). This impact was stronger than nitrogen deficiency which reduced plant height by 7 cm (WW N0 compared to WW N+ conditions). As shown on yield, there was no significant difference between WD N+ and WD N0 plant height. Unlike yield components that constitute destructive and final measurements, the temporal evolution of plant height for each stress condition may be very informative of plant behavior, indicating at what time the stress starts to impact plant growth. Figure 11A suggests that nitrogen deficiency begun at the end of March (the green curve is lower than the other one). At the beginning of the wheat growth cycle, the GPAI made it possible to distinguish the WW and WD modalities. Looking at GPAI curves (Figure 11B), nitrogen impacted also wheat behavior just after stem elongation, in both conditions WW and WD. This kind of stress during stem elongation could explain why the impact on ear density is significant (Figure 10D).

Considering tensiometers at more than 120 cbar (Figure 7A) and the water stress factor impacting LAI simulated by CHN (Figure 9A), the lack of available water could affect the potential plant growth from the end of April. Two weeks later, just before the heading date, temporal height measurement showed a decrease of wheat growth due to drought stress (gap between WW N+ and WD N+, Figure 11A). According to this representation, water stress deficit begun in the middle of May (during heading stage) and so this is consistent with drought impact seen on thousand kernel weight (Figure 10C).

The Green Plant Area Index was calculated from the RGB cameras by using both nadir and angle view. It is an integrative trait linked to the LAI that makes possible discrimination between WW and WD modalities at the beginning of stem elongation and also between N+ and N0 modalities (Figure 11B) during stem elongation. Strangely, at the beginning of the wheat development, the WD modality had a higher GPAI than the WW modality. At the end of the cycle, GPAI measurements showed an entry in senescence earlier for modalities under water deficiency from mid-May, while WW modality entered senescence from end of May/early June. GPAI was also affected by the nitrogen input with lower GPAI in N0 modalities compared to the N+ ones, especially in WW conditions.

**FIGURE 12:**
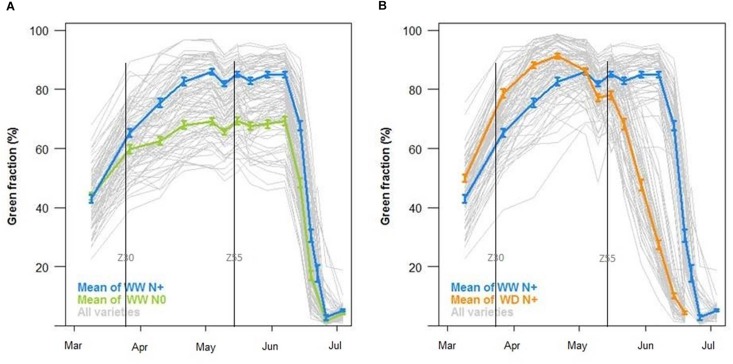
Green fraction (%) during the growing season **(A)** with the mean of well-watered conditions with nitrogen input (WW N, in blue) against well-watered conditions without nitrogen input (WW N0, in green) and **(B)** with the mean of well-watered conditions with nitrogen input (WW N, in blue) against water deficient conditions with nitrogen input (WD N+, in orange, *N* = 44, ±SE) and curves of all varieties (in gray).

Green fraction temporal evolution is also based on RGB Cameras data. Figure 12A shows a decrease of green fraction induced by nitrogen deficiency. The impact on bread wheat begun at the end of March (beginning of stem elongation), illustrated by the separation between WW N+ (blue curve) and WW N0 (green curve). The maximum level of green fraction decreased by 20% between the two treatments (Figure 12A). These observations are consistent with the impact of a nitrogen stress factor modeled by CHN (Figure 9B). Examining the water deficiency impact on green fraction, the maximum level was almost the same during the growing period, but the fall of green fraction began earlier on WD N+ (middle of May) than on WW N+ (middle of June) (Figure 12B). This could explain poor grain filling and smaller grain on WD than on WW (Figure 10C) and it is consistent with LIDAR, tensiometers and modeling with CHN. As blue, green and orange curves represent the mean data of each modality (respectively, WW N+, WW N0, and WD N+), gray curves represent all varieties, showing genetic variability.

**FIGURE 13:**
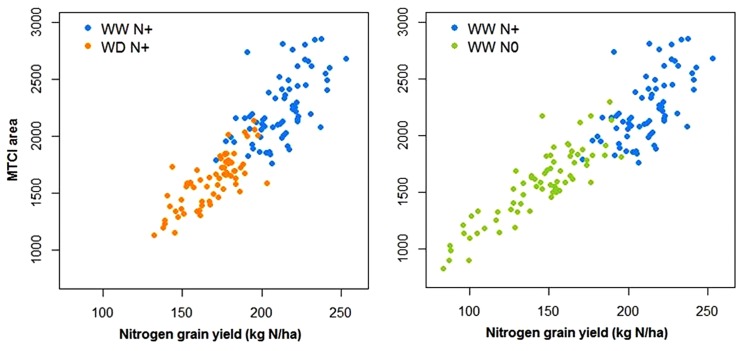
Relationship between the N grain yield and the area under the MTCI curve between flowering and maturity under well-watered conditions with and without nitrogen input (WW N+ and WW N0) and water deficient conditions with nitrogen input (WD N+, *N* = 66).

Using spectroradiometer data, as for other HTP variable acquired with time sequences, the area under the curve (AUC) of the MTCI index was calculated during grain filling (between flowering and maturity stage). Figure 13 shows that there was a positive and strong relationship between this trait and the quantity of nitrogen in the grain.

**FIGURE 14:**
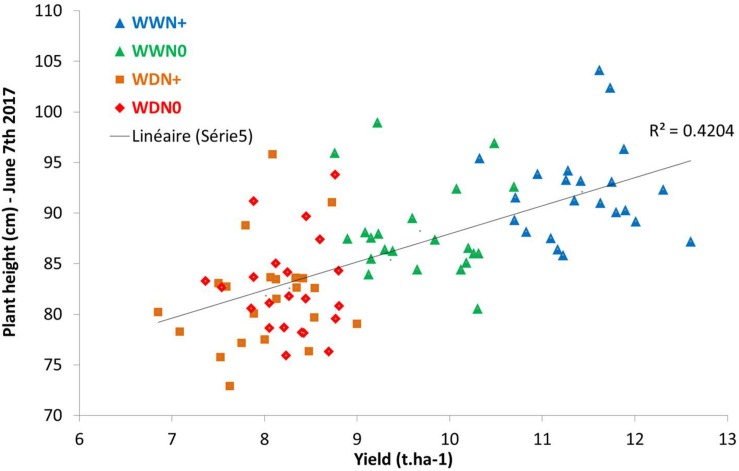
Correlation between yield (t.ha^–1^) and plant height during grain filling.

Looking at parameters of curves such as the maximum value, Figure 14 shows a correlation between the maximum height of the plant (at the beginning of grain filling) and yield. This linear correlation is lower at earlier stages.

**FIGURE 15:**
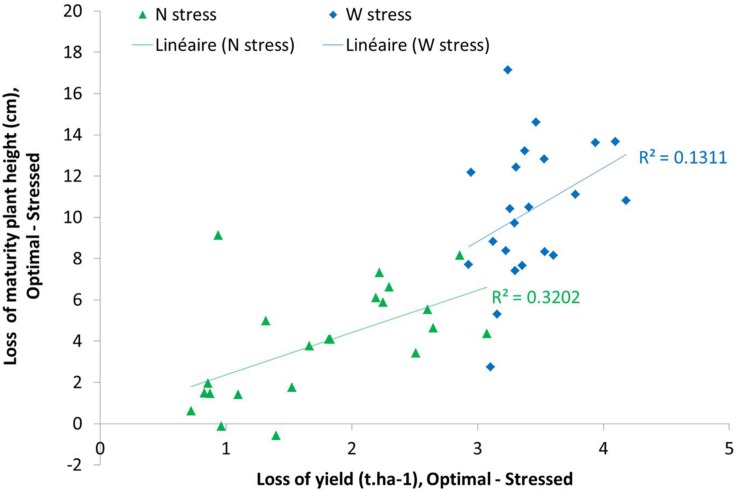
Mean of yield loss related to plant height loss at maturity stage between WW N+ and WW N0 (Nitrogen stress impact in green points) and WD N+ and WD N+ (Water stress impact in blue points).

Another way to analyze these data could be the difference between two modalities (optimal and stressed). In Figure 15, we could observe different behavior of varieties under nitrogen or water stress. This figure highlights the linear correlation between maximal plant height and yield under nitrogen and water stress. This correlation was lower in water stress condition than in nitrogen stress condition. We could also observe a higher diversity of varieties behavior under nitrogen stress than under water stress.

## Discussion

### Field Based Infrastructure Capabilities

PhénoField^®^ is a prototypical platform built in 2014. This field based phenotyping infrastructure (using rainout shelters, soil water sensors, and fine soil characterization) is offering various research possibilities on drought tolerance without other impact on the environment. Adjustments and development of methods are needed for its operation to better serve the needs of several research topics. Based on data acquired during 2017, we should be able to take into account experimental limits.

During BREEDWHEAT 2017, the control of water stress levels were allowed by the monitoring of soil water tension and soil humidity. It helps us to avoid plant water stress for WW conditions. In addition, the control system of the rainout shelters allowed automatic interception of up to 92% rainfall (Figure 4A). The remaining 8%, which was a very small amount of water, was probably due to the movement time from the garage position to the protection position of the shelters (although a slight inflow of water was detected on the edge of the first southwest plot of the protected area). Prevailing southwesterly winds seemed to cause 35 mm of precipitations under the first meter protected by the shelters, though without affecting the yield of this microplot-line. Based on these results, we could move the whole microplot positioning under the rainout shelter 40 cm forward to the northeast in order to avoid this small rainfall entry and fully protected the field trial.

During the raining period, crop protection by shelters induced a PAR decrease and an overall air temperature increase (Supplementary Figure S3). Such secondary effects were generally reported as being due to the use of shelters with values close to those reported by Paajanen et al. (2011) who noticed a 44% reduction in the PAR due to shelters. In this way, the automatic control of shelters limited these side effects by reducing the time of protection. The result acquired in 2017 was that the cumulative PAR decreased by 3.5% and the temperature increased by 1.8%. These results inspire us to continue measuring how shelters impact the environment over time and to use their automatic movement in order to reduce protection time. Moreover, these effects seem to be very low on wheat growth but the expected impact on other crops must be simulated using the check plots. That is why we should use the CHN model to simulate what could be the impact on other crops.

Mapping soil characterization is important to understand soil heterogeneity in order to take it into account in the interpretation of agronomic variables (Di Virgilio et al., 2007). The WHC map showed significant variability typical of the soil of this region. Indeed, the clay-silt soils developed on Beauce limestone have a variability of thickness within very short distances (sometimes metric, Seger et al., 2017). It is important to consider this variability during crop management because crops growing on limited WHC are more subject to drought and will probably require more irrigation. The simultaneous use of the WHC map, the CHN model, tensiometers and TDR probes allows monitoring and control of limiting factors applied to the crops. However, work should be carried out to characterize root depth in order to refine the map of the WHC, and so define a map of the water accessible to the plant. The ultimate goal is to access to microplot scale data to characterize crop water consumption for each microplot tested on PhénoField^®^.

### High Throughput Phenotyping to Characterize Drought and Nitrogen Stress Impact

PhénoField^®^ uses high throughput field based phenotyping development to characterize responses of crop to abiotic stresses. The example of the BREEDWHEAT field trial conducted in 2017 demonstrated the capacity of the system to characterize drought and nitrogen stress impact on wheat growth, with accuracy needed to differentiate treatments like wheat varieties.

In this trial, water and nitrogen deficiency have different impact on agronomical traits but we have a significant difference between varieties on all agronomical traits (Supplementary Table S2).

In our case, the application of drought during the stem elongation period (Figure 8) negatively affected the yield of the wheat (Figure 10). Drought continued to severely affect yield during grain-filling, inducing earlier green fraction decreasing that could be considered as an indicator of earlier leaf senescence (Figure 12). Drought also leads to a significant reduction in the thousand kernel weight (Figure 10). Such results are often reported and well described in literature (e.g., Estrada-Campuzano et al., 2008) with effects depending on the stress intensity and on the plant-growth stage at the time of application (Fahad et al., 2017). In the same way, induction of nitrogen deficiency led to a reduction in the yield compared to the optimum nitrogen conditions (at WW conditions). It is also consistent with literature relating the yield loss to a diminution of ear density (e.g., Le Gouis et al., 2000). The performance of different bread wheat varieties for yield and yield components varied and could be relatively contrasted in similar conditions. Indeed, it should be interesting for variety characterization to observe the impact of these differences on grain yield.

Differences linked to stress conditions were also noticed with the embedded sensors LIDARs, RGB cameras and spectroradiometers. First of all, these sensors provided temporal information on wheat varieties and crop management systems. The height calculated with the LIDARs could be a good indicator of the onset and magnitude of the plant’s stress (Madec et al., 2017) but also of the date of the starting point of stress impact. Similarly, the RGB cameras enable estimations of the GF (Figure 12) and GPAI (Figure 11B). The GPAI has been reported to be a relevant variable for several key processes involved in canopy functioning (Baret et al., 2010), especially the stay-green of the LAI which participates in biomass accumulation. It is acknowledged that wheat genotypes that sustain flag-leaf photosynthesis for longer periods produce better yields (Christopher et al., 2008); therefore, the start and rate of senescence of flag leaves in wheat can be used as an indicator of stress resistance for determining resistance to stress (Larbi and Mekliche, 2004). PhénoField^®^ sensors and the actual processing data are not allowing us to look at flag leaf alone but rather the plot canopy. Here, nitrogen deficiency had a negative effect earlier than drought as shown on GPAI (Figure 11B) but also on plant height (Figure 11A). Strangely, at early stage (end of winter), the WD modality had a GPAI higher than that of the WW modality (Figure 11B). A lack of nitrogen probably link to irrigation on WW and a loss due to nitrogen leaching could explain the effect on GPAI of the latter modality. The spectroradiometer data are currently used to extract common vegetation indexes (as NDVI, MCARI2, or MTCI). These vegetation indexes are complementary and used differently depending on the phenological stage. They can be related to yield components and nitrogen content (Dash and Curran, 2004; Walsh et al., 2018) as it was reported here with the correlation between the MTCI AUC and the nitrogen grain yield (Figure 13). Such indexes could, as the MTCI, discriminate the response of wheat varieties to stress and so, explain varietal behaviors (Basso et al., 2016).

In addition to temporal analyses, curves parameters whether they are directly read on a drawn curve direct or calculated from a fitted curve, could also be an alternative way of analyzing data in high throughput phenotyping systems. Nevertheless, stress indicators such as areas under the curve shown previously, appear to be more relevant than using point values to explain performance from high-throughput phenotyping data.

It seems that these variables that describe the behaviors of varieties will enrich breeding methodologies in order to accelerate genetic progress especially given the predictions that climate change will bring about more drought and heat stress in the majority of wheat environments (Barros et al., 2014). High throughput phenotyping (HTP) is particularly fast compared to manual measurements and provides a non-destructive method for accessing physiological and biochemical trait responses to environmental conditions (Araus et al., 2018) at each development stage. The development of the HTP system has originally focused on measurements of large numbers of plants in controlled environments. This approach provided advanced knowledge of the plants’ physiological processes. Nonetheless, studying plant responses in controlled environments representing field environments has well-known limitations (White et al., 2012). So, developing a field platform like the PhénoField^®^ platform represents a novel type of tool dedicated to assess the responses of crops to stress scenarios by using the HTP techniques in the field.

## Conclusion and Outlook

As shown with 2017 dataset, the automated movement of the 8 rainout shelters demonstrated its performance to control water and nitrogen deficiency on bread winter wheat field trials without other significant impact on the trial environment. This data set is limited on genotype number to perform 3 replicates and 4 stress conditions. PhénoField^®^ platform, with its large plot capacity, could also provide genetics’ field trials with more than 300 genotypes under sole drought conditions and enhance knowledge on physiological analyses, varieties tolerance evaluation or genomic regions controlling these complex traits. 2017 was the 1st year using field HTP with a data-processing pipeline that still has to be improved. However, it showed promising results, especially with the dynamics of sensor traits allowing the calculation of relevant indicators of abiotic stresses.

The actual set of sensors allows testing of many traits and new parameters to select the best way to discriminate modalities as varieties under nitrogen or water deficiency. The possibilities to analyze the 2017 data set are really important and we are aware that only a small part was explored in this paper. Nevertheless, our first objective was to demonstrate the PhénoField^®^ platform’s capacity to efficiently conduct the test protocols (intensity and duration of stress). Conducting trials on field HTP induced significant soil environment genotype interactions and, mapping the spatial variation of soil characteristics (as well as WHC soil resistivity) is essential for incorporating field variability into crop management and for the interpretation of experimental results. Acquisition of field phenotyping data is now a well-established process, allowing weekly data registration from spectroradiometers, LiDARs and RGB cameras. Acquisition of measurements for the entire platform amounts to about 100 GB per day and only a minor part of it is used to calculate the height, GPAI or vegetation index presented here. In future publications, we will explore more in details curve parameters in regards to agronomic data. Moreover, since 2018, the inversion of radiative models available in PhénoField^®^ data processing chain allows access to the chlorophyll content, which is even more relevant than the vegetation indexes in qualifying nitrogen stresses. Indeed, the calculation of the commonly used vegetation indexes only used 1% of the available spectra and exploitation of other wavelengths could highlight other physiological processes (Araus et al., 2018).

PhénoField^®^ is also a platform acting as a field HTP reference used to calibrate and develop innovative phenotyping tools such as the set-up of new sensors. Thus, measurements by drones can be compared to the acquisition of gantries. Drones will allow complementary sampling to intercept diurnal variation and data acquisition faster than with embedded sensors and could be applied on multiple experimental sites. Alternatively, root phenotyping techniques such as Minirhizotrons, using scanner-based methods, could be tested in parallel with gantry measurements in order to access correlated traits between root and above-ground biomass.

Indeed, PhénoField^®^ is connected to other HTP platforms in order to enhance knowledge on genotyping using environment interactions. As part of the PHENOME-EMPHASIS/France network, PhénoField^®^ is highly connected to other French field platform like Pheno3C (INRA, Clermont-Ferrand), but also to controlled platform like PhenoArch (INRA Montpellier). Phénomobile which consists of field mobile HTP robots and ALPHI (A Light Innovant PHenotyping), a system using a boom and a tractor with inbuilt sensors, are also used in the same network^4^. Such connections between these tools involve a common sensor set, an identical processing data chain and a large amount of data to handle and offer huge possibilities for breeding or other research programs.

## Author Contributions

KB: platform management, measurement, and writing of the manuscript. FL: data analyses and writing of the manuscript. AF: measurement, system establishment, system discussion, and writing of the manuscript. CH: technical realization and measurement. MB: results and discussion. JL: results, discussion, and evaluation of the manuscript. BdS: system discussion. BP: database organization and model CHN discussion. ST: system processing data. J-PC: evaluation of the manuscript.

## Conflict of Interest Statement

The authors declare that the research was conducted in the absence of any commercial or financial relationships that could be construed as a potential conflict of interest.

## Footnotes

1 https://www.phenome-emphasis.fr/

2 https://breedwheat.fr/

3 http://www.irrinov.arvalisinstitutduvegetal.fr/irrinov.asp

4 https://www.english.arvalisinstitutduvegetal.fr/view-2158-arvstatiques.html
