# Supplementary material for: Management and Characterization of Abiotic Stress via PhénoField®, a High-Throughput Field Phenotyping Platform
Source: Front Plant Sci. 2019 Jul 16;10:904. doi: 10.3389/fpls.2019.00904 (PMC6646674; doi:10.3389/fpls.2019.00904)
Supplement: Supplementary file 1 [file Data_Sheet_1.docx]

Supplementary Material

Management and characterization of abiotic stress via PhenoField®, a high-throughput field phenotyping platform.

Katia Beauchêne^1*^, Fabien Leroy^1^, Antoine Fournier^1^, Céline Huet^1^, Michel Bonnefoy^1^, Josiane Lorgeou^2^, Benoît de Solan^2^, Benoît Piquemal^2^, Samuel Thomas², Jean-Pierre Cohan^3^

^1^ARVALIS – Institut du végétal, Ouzouer-Le-Marché (41), France

^2^ARVALIS – Institut du végétal, Boigneville (91), France

^3^ARVALIS – Institut du végétal, La Chapelle Saint Sauveur (44), France

***Correspondence:**

Author for correspondence,

Tel: + 33 2 54 82 33 17,

Fax: +33 02 54 82 33 11,

email: [k.beauchene@arvalis.fr](mailto:k.beauchene@arvalis.fr)


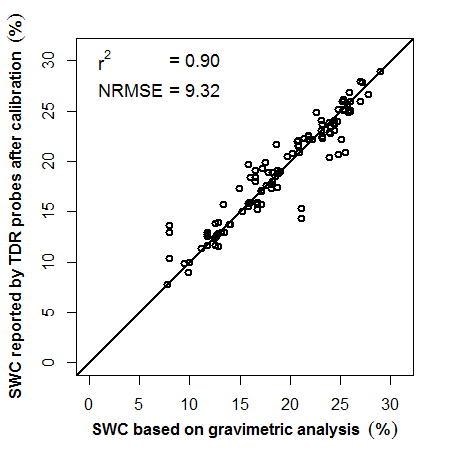

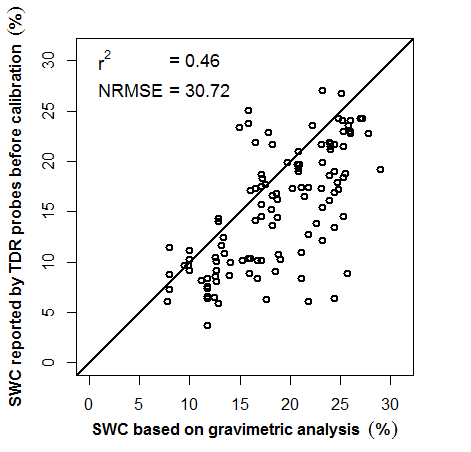


**a)**

**b)**

**Supplementary Figure 1.** Relationship between the soil water content (SWC) obtained with gravimetric measurements and measured with TDR probes before (a) and after (b) their calibration.


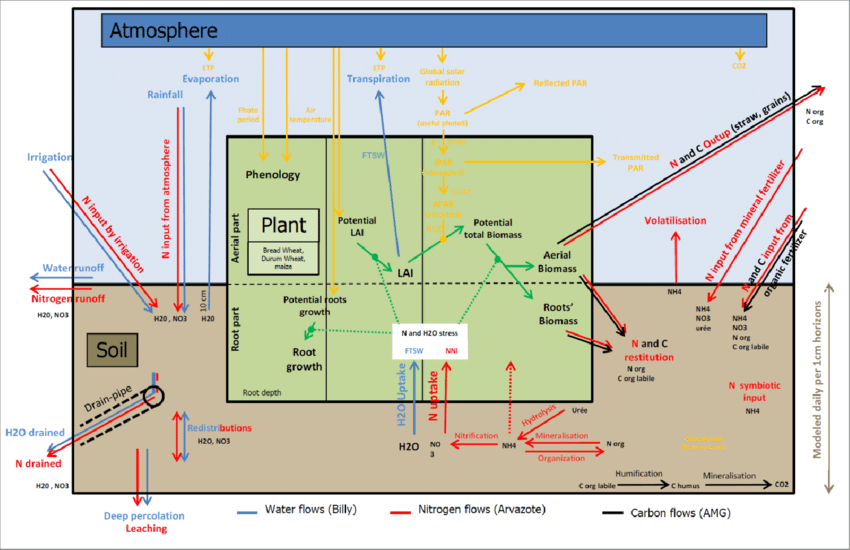


**Supplementary Figure 2:** ‘CHN” model synopsis (from Soenen et al., 2016)


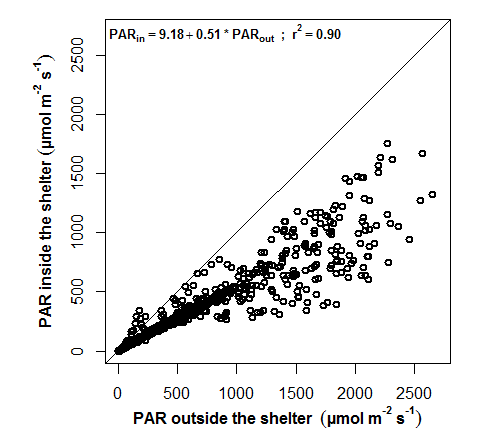

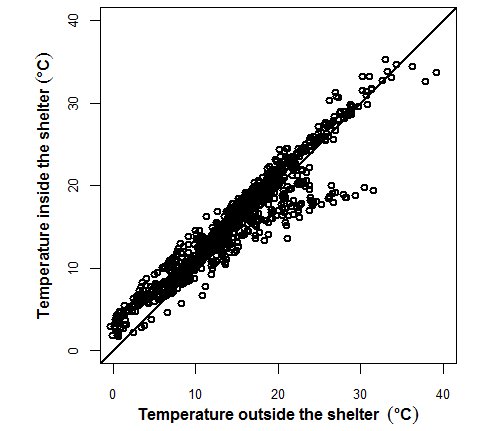


**b)**

**a)**

**Supplementary Figure 3:** Photosynthetic Active Radiation (PAR) (a) and air temperature (b) measured inside and outside the shelters during crop protection in 2017.

**Supplementary Table I:** Cultural practices and application of abiotic spring stress on wheat during Breedwheat experiment in 2017

|  |  | Oct. | Nov. | Dec. | Jan. | Feb. | Mar. | April | May | June | July | Total |
| --- | --- | --- | --- | --- | --- | --- | --- | --- | --- | --- | --- | --- |
| Rain (mm) |  | 8.8 | 52.6 | 16.8 | 23.8 | 43.4 | 59.2 | 13.6 | 68.0 | 49.6 | 9.4 | 345.0 |
| Rain interception | WW  (N+ and N0) |  | 19 | 0 | 7.8 | 1.6 |  | 9.4 | 22 | 42.8 | 9.4 | 112 |
|  | WD  (N+ and N0) |  | 19 | 0 | 7.8 | 17.4 | 59.2 | 13.6 | 68 | 49.6 | 9.4 | 244 |
| Irrigation | WW  (N+ and N0) |  | 40 | 40 |  |  | 15 | 15+35 | 30+30+20 | 20 |  | 115 |
|  | WD  (N+ and N0) |  | 40 | 40 |  |  | 15 | 15 |  |  |  | 110 |
| Total Water supply (mm) | WW  (N+ and N0) | 8.8 | 73.6 | 56.8 | 16 | 41.8 | 74.2 | 54.2 | 126.0 | 26.8 | 0 | 478.2 |
|  | WD  (N+ and N0) | 8.8 | 73.6 | 56.8 | 16 | 26.0 | 15 | 15 | 0 | 0 | 0 | 211.2 |
| Nitrogen fertilization  (kgN/ha) | N+  (WW and WD) |  |  |  |  |  | 82 | 50 |  |  |  | 132 |
|  | N0  (WW and WD) |  |  |  |  |  |  |  |  |  |  | 0 |

**Supplementary Table II:** Statistical effect of water condition (“Water”), nitrogen supply (“Nitrogen”) and variety and their interactions on yield, grains protein concentration, Thousand kernel weight, Plants density , Grains.m2 calculated with three-ways ANOVA.s and expressed as P-value scale : *** (p <0.001) ; ** (0.001<p<0.01) ; * (0.01<p<0.05) ; (*) (0.05<p<0.1) ; ns = non significant

|  | |  | Significance | | | | | | | |  |
| --- | --- | --- | --- | --- | --- | --- | --- | --- | --- | --- | --- |
|  |  | | Water | Nitrogen | Variety | Water  *Nitrogen | Water  *Variety | Nitrogen  *Variety | Water  *Nitrogen  *Variety |  |  |
|  | | Yield | 0.0148 | 0.0653 | 1.39e^-07^ | 0.0274 | 0.6031 | 0.0754 | 0.3525 |  | |
|  | | Grain protein concentration | 0.0063 | 4.620e^-05^ | 3.082e^-14^ | 0.02040 | 0.0278 | 0.03166 | 0.1821 |  | |
|  | | Thousand kernel weight | 0.0570 | 0.0002 | < 2.2e^-16^ | 0.1084 | 2.522e^-07^ | 5.926e^-06^ | 0.0506 |  | |
|  | | Plants density | 0.2675 | 0.3604 | 9.5e^-08^ | 0.3137 | 0.8571 | 0.3874 | 0.6295 |  | |
|  | | Grains.m^2^ | 0.0220 | 0.0023 | < 2.2e^-16^ | 0.0054 | 0.8179 | 0.2284 | 0.45615 |  | |

|  | |  | Significance | | | | | | | |  |
| --- | --- | --- | --- | --- | --- | --- | --- | --- | --- | --- | --- |
|  |  | | Water | Nitrogen | Variety | Water  *Nitrogen | Water  *Variety | Nitrogen  *Variety | Water  *Nitrogen  *Variety |  |  |
|  | | Yield | * | (*) | *** | * | ns | (*) | ns |  | |
|  | | Grain protein concentration t | ** | *** | *** | * | * | * | ns |  | |
|  | | Thousand kernel weight | (*) | *** | *** | ns | *** | *** | (*) |  | |
|  | | Plants density | ns | ns | *** | ns | ns | ns | ns |  | |
|  | | Grains.m^2^ | * | ** | *** | ** | ns | ns | ns |  | |
